# Older Adults Encode Task-Irrelevant Stimuli, but Can This Side-Effect be Useful to Them?

**DOI:** 10.3389/fnhum.2020.569614

**Published:** 2020-10-29

**Authors:** Zsófia Anna Gaál, Boglárka Nagy, Domonkos File, István Czigler

**Affiliations:** ^1^Institute of Cognitive Neuroscience and Psychology, Research Centre for Natural Sciences, Budapest, Hungary; ^2^Doctoral School of Psychology (Cognitive Science), Budapest University of Technology and Economics, Budapest, Hungary; ^3^Institute of Psychology, Eötvös Loránd University, Budapest, Hungary

**Keywords:** aging, N170, LPC, old/new effect, ERP

## Abstract

We studied whether, due to deteriorating inhibitory functions, older people are more likely to process irrelevant stimuli; and if so, could they later use this information better than young adults. In the study phase of our experiment, a Posner-type gaze-cued version of a Simon task was performed in which we presented task-irrelevant cues, where faces or patches with either left- or right-looking dots for the pupil of the eye preceded the task to press a button congruent or incongruent with the presentation side of the target stimulus. In the follow-up test phase, participants completed an unexpected facial recognition test. In the study phase not only a decreased P1, but also an increased N170 amplitude of the event-related potentials (ERPs) were found in older, compared to younger adults, and also for faces compared to patches. Even though in the test phase both age-groups could recognize the faces better than statistically by chance, neither the older nor the younger participants could discriminate them effectively. The late positive component (LPC)—the ERP correlates of the old/new effect, being the higher amplitude for the earlier presented stimuli when compared with the unseen stimuli during the recognition test—was not evolved in the older group, while a reversed old/new effect was seen in younger participants: higher amplitude was found in *New-Right* and *Old-Wrong* conditions (for faces they did not recognize independent of seeing them before) compared to *Old-Right* and *New-Wrong* conditions (for faces they thought they recognized from the study phase). In conclusion, although older adults showed enhanced processing of task-irrelevant stimuli compared to younger adults, as indicated by the N170 amplitude, however, they were not able to utilize this information in a later task, as was suggested by the recognition rate and LPC amplitude results.

## Introduction

It is a general fact that some cognitive performances decline with age—one of the most affected areas being working memory. The reasons for compromised working memory are the deterioration of the prefrontal cortex (West, 1996; Tisserand and Jolles, 2003), and a decrease in the efficiency of inhibitory processes (Hasher and Zacks, 1988). According to the load theory of attention (Lavie, 2005) where there is insufficient cognitive control, distractors will be processed to a greater extent, causing older adults to encode more irrelevant elements, which interferes in their working memory. The question that interested us was whether older adults encode task-irrelevant stimuli, and if so, can this side-effect be useful to them.

In a relevant experiment (Biss et al., 2013), participants had to study and recall a list of words. In the second phase, a 1-back task, half of the listed words were used as distractors. In the final phase, participants were asked, in a surprise test, to recall the original list of words again. Younger adults were found to forget both the repeated and unrepeated words at similar rates, whereas the older adults forgot the unrepeated words, but recalled the 1-back distractors at a higher rate. Biss et al. (2013) considered that exposure to distracting elements was a repetition of these stimuli which were reinforcing the memory trace in older adults who could not eliminate the task-irrelevant words in the second phase. Similar results were found in the same lab by Weeks and Hasher (2018): older adults showed a greater priming effect than younger ones for words that were used as distractors in an earlier task; whilst no age-related differences were evident in priming for target (attended) pictures.

The goal of our current study was to repeat these results of enhanced coding of irrelevant elements with non-verbal stimuli in older adults using task-irrelevant faces. Two questions we faced were: do older adults process the task-irrelevant stimuli to a greater extent than younger ones; and can they use this information in a subsequent task? In attempting to answer these questions we registered the event-related potentials (ERPs) to track face perception (N170) and recognition (ERP old/new effect).

Event-related potentials are good tools to reveal the effects of unattended stimuli where the participants do not otherwise display behavioral answers. In our study, we tracked the processing of irrelevant faces by focusing on the N170 ERP component. The N170 is a negatively deflecting component that peaks at around 170 ms after stimulus onset above the occipito-temporal brain areas. Its amplitude is larger for faces when compared with objects, with the former having a right hemisphere distribution, and the latter a bilateral scalp distribution (Bentin et al., 1996; Rossion, 2014). Neuroimaging studies have revealed a distinct neural network in connection with face processing which includes both the fusiform and the occipital face area along with the superior temporal sulcus (Haxby et al., 2000). Concerning face-specificity: Itier and Taylor (2004) recorded ERPs when participants viewed not only upright and inverted faces but also other objects (like houses or flowers), which revealed a significantly shorter N170 latency for the upright faces and a larger amplitude for both the upright and inverted faces compared to the other object categories. Additional ERP analyses verified that N1—originally evoked by objects around the same time window as N170 but more occipitally and medially—and N170 are distinct and qualitatively different components; while N1 may represent a return to baseline from P1, the N170 has an extraneural generator for face processing. Moreover, this study examined P1 components as well, and observed a delayed and larger P1 for faces rather than other objects, and in particular for inverted rather than upright ones, which could be a consequence of low-level feature differences. They concluded that the P1 component was an early global response for the holistic characters of face stimuli, while the N170 component reflected the processing of face configuration and the relationship of facial features to each other.

Age-related studies found both higher N170 amplitude in the older compared to younger adults (Chaby et al., 2003; Gao et al., 2009; Daniel and Bentin, 2012), and no age-group differences (Pfütze et al., 2002); and also, the latency was not affected by aging (Chaby et al., 2003). It was a general finding that the older group did not show any asymmetric scalp distribution (Pfütze et al., 2002; Chaby et al., 2003; Gao et al., 2009; Daniel and Bentin, 2012; Limbach et al., 2018).

In the second step, we wanted to test whether there is an accessible memory trace in a later task. For this purpose, we were able to use the old/new effect of the recognition test (Wilding and Rugg, 1996; Friedman and Johnson, 2000). In this test, participants have to decide whether they have seen the given stimulus in an earlier task. The ERP of the “old” (earlier presented) and “new” (not earlier presented) stimuli show differences in two components. The first being the early old/new effect (FN400) which is within the range of 300–500 ms after stimulus onset and has a fronto-central or left frontal scalp distribution. This component is evoked by those items which were correctly identified as being “old,” and from earlier research, this has been associated with familiarity (Curran, 2000; Paller et al., 2007). The second component, the late old/new effect (LPC—analyzed in this study) peaks around 500–800 ms, and has a centro-parietal, or left parietal maximum. It is generally thought that this is the index for recollection—recognition accompanied by accurate source memory—as the correctly categorized old stimuli evoke a more positive-going deflection than the new elements (Paller and Kutas, 1992; Rugg and Curran, 2007; Zheng et al., 2016).

In our experiment participants had to perform a Simon task, and press the left/right button on a keyboard when a letter B/J was presented on the left or right side of the monitor. The side of the target stimulus corresponded (congruent condition) or not (incongruent condition) to the required responding hand. Target stimuli (B/J) were preceded by either a face or a patch stimulus with a sideward gaze. We found that the task-irrelevant gaze influenced the performance of the older but not the younger adults, adding a further load on their cognitive processing (Nagy et al., 2020). In the present study, we investigated whether older adults could process these faces deeper than younger adults. We hypothesized that a larger N170 component will result in the older adults being able to encode irrelevant face stimuli better than younger adults; and as a consequence of this with their deeper processing of unattended face cues (Eimer, 2000; Holmes et al., 2003), this would assist them to recognize the earlier presented faces more effectively, as indexed by the number of hits and the ERP old/new effect in the recognition test.

## Materials and Methods

Participants and the study phase of the experiment have been published in Nagy et al. (2020).

### Participants

In the experiment 24 younger (mean age: 22.0, SD = 2.3, range 18–27 years; 12 females) and 21 older adults (mean age: 68.1, SD = 3.25, range: 67–74; 10 females) participated. One of the older participant’s data was omitted for technical reasons. We ruled out dementia-related differences by full-scale Wechsler IQ (measured by the Hungarian version of WAIS-IV, Rózsa et al., 2010); *IQ*_(younger group)_ = 107.7 ± 16.6, *IQ*_(older group)_ = 128.9 ± 15.3. Every participant was right-handed, in addition to having a normal or corrected-to-normal vision, they had no history of any kind of neurological or psychiatric disorder; and all of them were paid for their contribution.

**Figure 1:**
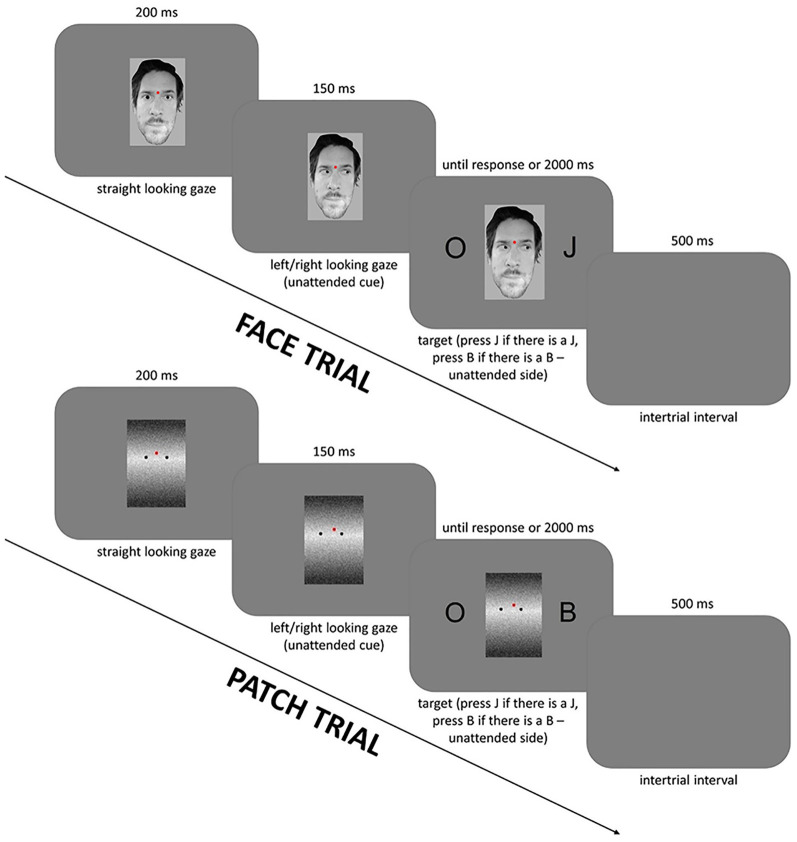
Experimental design: the sequence and timing of stimuli in the face cued trials (top row) and the patch cued trials (bottom row). The figure is similar but not identical to the original image and is therefore for illustrative purposes only.

The protocol was approved by the Joint Psychological Research Ethics Committee (EPKEB, Hungary), and written informed consent was obtained from all participants as well as a separate consent from the individual in Figure 1 for the publication of any potentially identifiable images or data included in this article.

### Procedure

The experiment had two phases. In the first (study) phase the participants executed a Posner-type gaze-cued version of a Simon task (Nagy et al., 2020), and in the second (test) phase they completed a recognition test. The experimental stimuli were presented with MATLAB R2016b (The MathWorks, Inc., Natick, MA, USA) using a 19 inch CRT monitor (LG Flatron F920B, 75 Hz refresh rate) from 1.2 m distance.

The study phase trials (Figure 1) started with first a task-irrelevant cue—a straight-looking face or a patch on a gray background (unattended/distracting stimulus) with a red fixation dot which was presented for 200 ms. This was then followed by a left- or right-looking gaze, which appeared for 150 ms and was followed by the Simon task stimuli—two symmetrically positioned black letters at the left and right side of the gazing face or patch stimulus. These letters were presented for as long as it took the participants to respond, or up to, but not beyond, 2,000 ms. One of the letters was always an O and the other letter was either B or J. Participants had to press the left or right button when they saw the letters B or J, respectively. The size of the letters was 1.4° × 1.3° visual angle, the size of the faces was 3.3° × 4.1° from a viewing distance of 120 cm. After every response, a blank gray background was presented as an inter-trial interval for 500 ms. The session started with a practice block of 50 patch trials. The patch was a control for the face. Half of the participants started with either face or patch condition; and both conditions contained eight blocks of 50 trials making 400 trials in total. In the face condition, 50 individual faces were shown, and these were repeated between subsequent blocks; and also the participants were not prompted to recognize these faces later. More detailed information and results for the Simon and gaze cueing effect can be found in Nagy et al. (2020).

In the test phase, which directly followed the study phase without a break, every participant executed a recognition test in which 50—either new faces (not seen before) or 50 old faces (seen earlier)—were presented separately, making in total 100 trials with 100 faces. For each trial, a face stimulus was shown on gray background for 2,000 ms, or until a response; and in the interval of 500 ms between each trial there was a blank gray background. The old and new faces appeared randomly, and the participants had to press either the B, left-handed or the J, right-handed buttons (in the position of letters A and L on a QWERTY keyboard), following whether or not they recognized the face from the study phase of the experiment.

The faces in these experiments were collected from online free sources, and were all male, frontal views, with either neutral or slightly smiling expressions. Corel Photo-Paint X3 was used to convert them to black and white images, and to create their left- and a right-gazing appearance by modifying the position of the pupils. In the patch condition we used 55 squares of different sizes (3.7 × 6.7 px − 19 × 53 px) and colours [rgba(36, 36, 36, 1) − rgba(249, 249, 249, 1)] per patch, placed in a random but not face-like order. Additionally, we applied Gaussian blur to every square and set the luminance to be similar to the face images. Two black dots were placed in the same position as the eyes and the left/right-looking gaze was imitated by moving these two dots correspondingly. Both face and patch images were seen under 3.3° × 4.1° visual angle, and a red fixation point was placed between the eyebrows on the face images, and in the same position on the patch images.

### ERP Recording

EEG was recorded with NeuroScan 4.5 recording system (NeuroScan SynAmps2 amplifier, USA, Brain Products EasyCap, Ag/AgCl electrodes, DC-200 Hz, sampling rate: 500 Hz). We used 28 locations following the extended 10-20 system: F7, F3, Fz, F4, F8, FC3, FC4, T7, C3, Cz, C4, T8, CP5, CP6, P7, P3, Pz, P4, P8, PO7, PO3, POz, PO4, PO8, O1, Oz, O2, and with AFz as ground, and the reference on the tip of the nose. Vertical and horizontal eye movements were recorded by AF7, and the electrodes were placed below the left eye (VEOG) and in the outer canthi of the eyes (HEOG). The impedance of the electrodes was kept below 10 kΩ.

### Data Analysis

#### Behavioral Data

We measured the rate of correct responses (hit and correct rejection) and erroneous responses (miss and false alarm) for the old and new faces within the critical response time of 2,000 ms, where hit/miss is a yes/no response to an old face, and false alarm/correct rejection is a yes/no response to a new face. To consider the guessing rate we applied the Two-High Threshold Model correction suggested by Snodgrass and Corvin (1988). Accordingly, the discrimination index—the probability that an old/new face will exceed the old/new recognition threshold—is (Pr = HR − FAR), where HR is the hit rate, and FAR is the false alarm rate. The bias index (Br)—the probability of a yes response in the uncertain state—is calculated as Br = FAR/[1 − (HR − FAR)]. We also calculated median reaction time (RT) to balance out intraindividual variability which tends to be higher in older adults (Myerson et al., 2007; Whelan, 2008).

#### ERP Data

Offline EEG processing started with a non-causal Kaiser-windowed Finite Impulse Response filter with the parameters set at 30 Hz of cut off frequency, a beta of 12.2653, and a transition bandwidth of 10 Hz for the low pass filter; and for the high pass filter 0.1 Hz of cut off frequency, a beta of 5.6533, and a transition bandwidth of 0.2 Hz. Independent Component Analysis (ICA) was performed with EEGLAB and was applied to our filtered EEG data to reject eye-movement artifacts—such as blinking and looking aside.

Segmentation was performed for cue-locked (P1 and N170—study phase) and test stimulus-locked (LPC—test phase) ERP components from −100 to 1,000 ms relative to first (centrally gazing) cue onset and the appearance of the old/new face stimuli consecutively. Baseline correction (prestimulus interval) was executed and epochs were rejected from averaging if they had a voltage change larger than 100 μV, resulting in an average of 328 epochs per condition for the study phase, and 22 epochs per condition for the test phase.

N170 of the study phase was defined by searching for the largest negative peak within the 130–250 ms time window after straight-looking cue presentation at P7, P8, PO7, and PO8 electrodes, and amplitudes were calculated as the mean amplitude within ±10 ms around peak latency for each participant. To eliminate the possible age-related effect of the P1 component, we calculated the amplitude difference between P1 and N170 components (the P1 component was defined as being the maximal positive peak between 50 and 150 ms after the centrally gazing cue and the mean amplitude was calculated within ±10 ms around peak latency for each participant was calculated on PO7 and PO8 electrodes). For the LPC of the recognition test, we calculated the mean amplitude of the 500–700 ms time window locked to face stimuli presentation at the Pz electrode. The type of face stimuli (old/new) and response (right/wrong) resulted in four outcomes (*Test responses*): *Old-Right*, *New-Right*, *Old-Wrong*, and *New-Wrong*.

Statistical analyses were performed with Statistica 13 (TIBCO Software Inc., Palo Alto, CA, USA). Repeated measures of ANOVAs were calculated with *Age* (younger/older) as between-subject factor, and the within-subject factors were *Cue* (face/patch); *Hemisphere* (left/right); and *Position* (PO/P) for the cue-locked ERPs; while the within-subject factor was *Test response* for both RT and ERP analyses in the recognition test. The effect size was calculated as Cohen’s *d* for *t*-tests and as partial eta square ($\eta_{\text{p}}^{2}$) for ANOVAs. *Post hoc* analysis was performed using the Tukey HSD test. *T*-tests were carried out for detecting significant differences from the baseline while we were looking for the test stimulus-locked LPC.

## Results

### Study Phase

**Figure 2:**
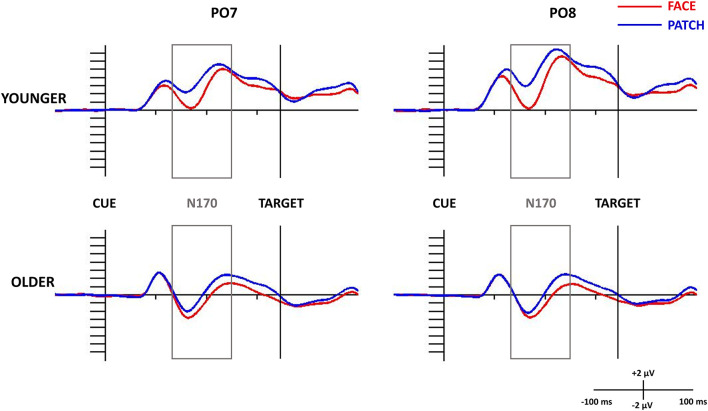
N170 component for task-irrelevant faces and patches in the study phase at the PO7 (left side) and PO8 (right side) electrode sites in younger (top panel) and older adults (bottom panel). The 0 ms time point on the x-axis is locked to the centrally gazing cue presentation and it represents the (−100, 500 ms) interval. ERPs for faces are shown in red, and patches in black. The gray rectangle shows the 130–250 ms time window in which we searched for the N170 component.

**Figure 3:**
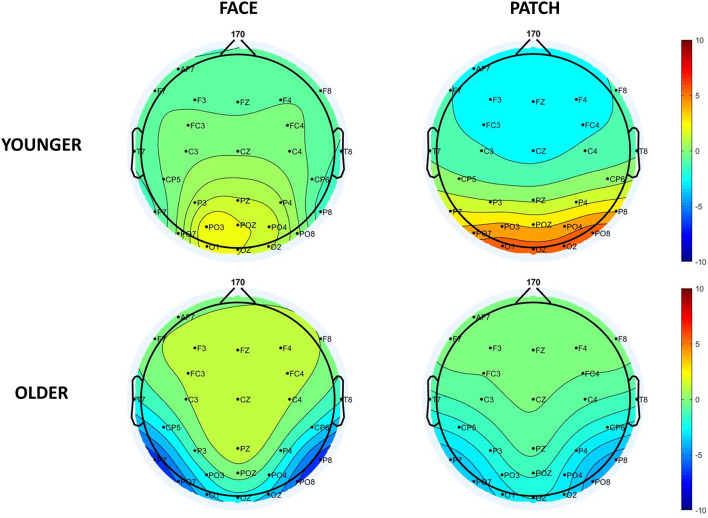
Scalp distribution of the N170 component for faces (left side) and patches (right side) in younger (top panel) and older adults (bottom panel).

When we examined both the P1 and N170 (Figure 2), we found *Cue* and *Age* main effects for the P1 amplitude, which was larger for patches compared to faces (*F*_(1,43)_ = 8.31, $\eta_{\text{p}}^{2}$=0.16, *p* = 0.006), and for younger compared to older adults (*F*_(1,43)_ = 8.80, $\eta_{\text{p}}^{2}$ = 0.17, *p* = 0.005), whereas the N170 amplitude was larger (by being more negative) for faces compared to patches (*Cue* main effect: *F*_(1,43)_ = 49.05, $\eta_{\text{p}}^{2}$ = 0.53, *p* < 0.001), and for older adults compared to younger ones (*Age* main effect: *F*_(1,43)_ = 22.49, $\eta_{\text{p}}^{2}$ = 0.34, *p* < 0.001). We also observed a *Cue × Age* interaction for the P1 component (*F*_(1,43)_ = 7.98, $\eta_{\text{p}}^{2}$ = 0.16, *p* = 0.007): its amplitude was higher for patches than faces in younger adults (*p* < 0.001), whilst no difference was found in the older group (*p* = 0.999), and younger participants had a higher amplitude for the patches (*p* = 0.007) and a tendentious difference for the faces (*p* = 0.096) when compared with the older adults. However, *Hemisphere’s* main effect or interactions were not found for the amplitude (for scalp distribution see Figure 3), and latency data did not reveal any differences in N170 latency between the groups and the conditions.

Similar to the N170 results the P1-N170 difference was also larger for face cues compared to patches (*Cue* main effect: *F*_(1,43)_ = 17.97, $\eta_{\text{p}}^{2}$ = 0.29, *p* < 0.001) and in older compared to younger participants (*Age* main effect: *F*_(1,43)_ = 4.19, $\eta_{\text{p}}^{2}$ = 0.09, *p* = 0.047).

### Behavioral Data—Test Phase

Recognition rates were low in both age groups. The hit rate was 0.47 (SD = 0.13) and 0.42 (SD = 0.13), whereas the false alarm rate was 0.30 (SD = 0.14) and 0.35 (SD = 0.15) in the younger and older groups, respectively. In the younger group the discrimination index was *P_r_* = 0.17, and in the older group *P_r_* = 0.07 with the two groups differing significantly (*t*_(43)_ = 2.30, *p* = 0.026). Despite the very low values, the discrimination indices significantly differed from chance (when *P_r_* = 0) in both age groups (younger adults: *t*_(23)_ = 5.15, *p* < 0.001; older adults: *t*_(20)_ = 2.91, *p* = 0.009). The bias index (B_r_) was 0.38 in the younger, and 0.37 in the older group, and there was no difference between the two age-groups (*t*_(43)_ = −0.33, *p* = 0.741). Concerning these findings, both groups used a conservative bias.

### Late Positive Component (LPC)—Test Phase

**Figure 4:**
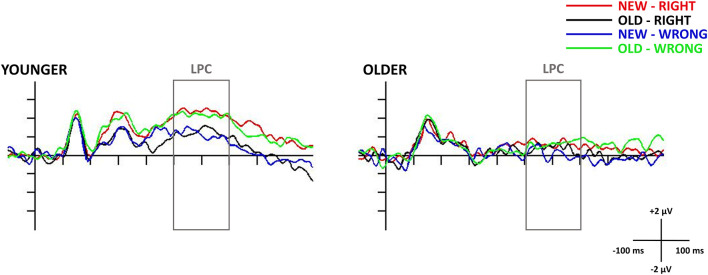
Test stimuli-locked late positive component (LPC) at the Pz electrode site in younger (left side) and older adults (right side). The 0 ms time point on the x-axis is locked to the test face presentation and it represents the (−100, 1,000 ms) interval. The presented waveforms are following the test responses (presented face—new/old and the participant’s response—right/wrong) and shown by the following colors: New-Right (red), Old-Right (black), New-Wrong (blue), Old-Wrong (green). The gray rectangle shows the 500–700 ms time window in which we searched for the LPC.

**Figure 5:**
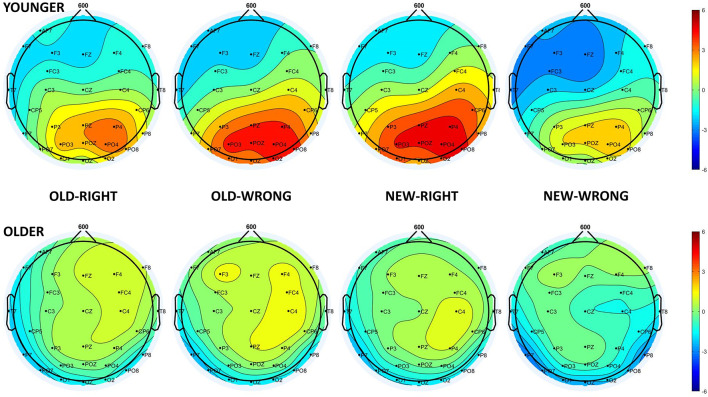
Scalp distribution of the LPC in younger (top panel) and older adults (bottom panel) for the four test responses in the following order: Old-Right, Old-Wrong, New-Right, New-Wrong.

In the older age group, the one-sample *t*-test showed no deviation from the baseline in the 500–700 ms time window, suggesting the absence of the LPC. However, in the *t*-test for the younger age group, there was a long-lasting positive difference from the baseline in the 400–800 ms time window, where LPC could be detected, in all four of the test responses. As a result of this, we were only able to consider the younger participants’ LPC data in any further analysis. The one-way repeated measures of ANOVA revealed a significant *Test response* main effect (*F*_(3,69)_ = 4.23, $\eta_{\text{p}}^{2}$ = 0.16, *p* = 0.008), and the Tukey HSD *post hoc* test showed larger amplitude for *New-Right* than for *New-Wrong* responses (*p* = 0.033), in addition to showing a tendency for a larger amplitude for *New-Right* compared with *Old-Right* (*p* = 0.060) and *Old-Wrong* compared with *New-Wrong* (*p* = 0.078) responses. All in all, there was a tendency for younger adults having larger LPC amplitudes for those responses where they made a “new face” decision (when they did not recognize the face from the study phase—*New-Right*, *Old-Wrong*)—compared to where they made an old face decision (when they thought they recognized the face from the study phase—*Old-Right*, *New-Wrong*, Figures 4, 5).

## Discussion

We were looking to see if the encoding of irrelevant items can be turned into an advantage by the elderly, so we used task-irrelevant face stimuli to study whether less effective distractor rejection can result in greater processing of these stimuli in older adults as we had hypothesized in line with the load theory of attention (Lavie, 2005) and the inhibitory control theory (Hasher and Zacks, 1988). As we had predicted, older adults could not effectively ignore the irrelevant stimuli, with the result that they processed the faces more deeply than young adults; though, despite this, they could not later use this information in a subsequent task.

In determining whether or not processing of irrelevant stimuli and consequently face discrimination occurred, we used a Posner-type gaze-cued version of a Simon task, in which faces and patches cued the task. These stimuli were presented in the center of the monitor during the whole of the trial, and every face was presented eight times during the experiment. The participants were told to ignore these cues, as there was no task relating to them. We found, similar to previous studies (Gazzaley et al., 2005; de Fockert et al., 2009), that our participants could not entirely ignore the irrelevant information: therefore the amplitude of the N170 component was higher for faces than for patches, which indicated that face discrimination had occurred.

As we hypothesized, we were able to find age-related differences: older adults had greater difficulty in preventing themselves from the processing of task-irrelevant stimuli, and the higher amplitude of the N170 component indicated that they were encoding the faces more readily than younger adults. Similar results were found by de Fockert et al. (2009) who presented target names along with distractor faces to-be-ignored. They registered the N170 component both in attended and not attended conditions, and their results showed no age-related differences in the attended condition but increased N170 in older compared to the younger adults in the unattended condition proving that age-related differences in the to-be-ignored condition reflected distractibility and not age-related differences in face processing.

While many studies have assumed that an increase in the interference can cause distractibility in old age by the unnecessary processing of irrelevant information, our N170 results give direct evidence to support this. Furthermore, not only were the faces processed to a higher extent by the elderly but their gazes as well, which influenced the older adults’ performance in the subsequent Simon task (Nagy et al., 2020). When the gaze focused on the target stimulus, the N2pc component indicated a visuospatial attention increase and a wrong-sided motor cortex activation—shown by the stimulus-locked lateralized readiness potential (s-LRP)—which was larger when compared to the incongruent gaze condition. Specifically, in the older group, we found that the congruent gaze (being the gaze is directed to the target) increased the reaction time and the error rate in the incongruent, but not in the congruent Simon condition by drawing attention from the side of the response, and this indicated an increased loading on their cognitive processing.

Besides we found N170 amplitude changes showed enhanced processing of the distractors, and also reduced P1 amplitude in older compared to younger adults, and for faces compared to patches. This reduced P1 was associated with increased attentional costs (Luck et al., 1994; de Fockert et al., 2009), which helped us to conclude that the faces distracted the participants more than the patches; and more importantly, the attention of the older compared to the younger participants was more distracted by task-irrelevant stimuli. It is worth mentioning that the P1 component can be modulated by low-level sensory information as well, like luminance, contrast, or noise (Schendan and Lucia, 2010; Rossion and Caharel, 2011). We also measured the P1-N170 amplitude differences to control the P1 effect, which similarly confirmed the results of the N170 component.

Even though the elderly appeared to process the faces more deeply than the younger adults, they still could not use this information effectively in a later task, in which they had to decide whether they had seen the presented faces earlier, and they had even a lower discrimination index as compared to the younger group. Behavioral results of the forced-choice test showed that although they could recognize the faces better than by chance, these values are too low to represent appropriate discrimination (participants with discrimination index lower than 0.2 were excluded from earlier studies, as it can be questioned whether they remembered to the earlier presented items, e.g., MacLeod and Donaldson, 2017). In the face-locked ERP of the test phase, the LPC was not seen in older adults, which indicated that they had not retained any representation of the faces.

On the surface, these results seem to be inconsistent with Biss and her colleagues’ findings, where they used task-irrelevant words (Biss et al., 2013) or names (Biss et al., 2020) superimposed on target pictures of the 1-back task, which served as an opportunity to reinforce the representation; and hence, to improve the performance in a later task. An important difference between Biss’s experiment and ours was that their participants first intentionally studied and recalled the words or names, developing semantic codes, and in the later phase this representation was consolidated by incidental rehearsal; maintenance rehearsal primed the existing representations. Other studies applying incidental learning direct attention to the later to-be-retrieved stimuli using tasks appropriate for deep (i.e., semantic) or shallow (i.e., perceptual) encoding (Wagnon et al., 2019), e.g., orientation judgment (upright/inverted) of face stimuli as shallow, and occupation judgment (actor/politician) as a deep incidental encoding of the faces (Marzi and Viggiano, 2010). However, in our case participants had no task at all with the stimuli they should have recognized later, there were no intention and motivation to learn these stimuli, there was no need for the deeper encoding of them, thus they did not have an initial representation that they could retain by stimulus repetition, and consolidate the memory trace. This difference enlightens an important point when we want to know, how to use incidental learning in older adults: an initial intentional attentional direction seems to be necessary if we would like to use distractor stimuli to improve memory, the encounter with the stimuli alone is not enough to be useful later.

Although we found the LPC in younger adults, the amplitudes did not show the usual order of the old/new effect, where the amplitude is higher for correctly categorized old stimuli compared to new items (Paller and Kutas, 1992; Rugg and Curran, 2007). In our study, the amplitude was more positive in *New-Right* and *Old-Wrong* compared to *Old-Right* and *New-Wrong* conditions—in cases participants thought they had not seen the face before compared to trials when they thought they had. Behavioral data showed that similar to older people, younger participants did not recognize the faces better than chance, i.e., they probably did not have the representations of these earlier successfully ignored faces. If incidental learning did not occur, the late positive component may not represent an old/new effect but seems to reflect the subjective probability of the stimuli (Horst et al., 1980), and greater attentional allocation occurred when the given face is thought to be new.

All in all, we can conclude that older adults not only pay more attention to task-irrelevant stimuli, but also process them more deeply; however, if these stimuli have not been introduced earlier, then the elderly do not form a long-term association, and hence cannot utilize this information in a subsequent task.

## Data Availability Statement

The datasets presented in this study can be found in online repositories. The names of the repository/repositories and accession number(s) can be found at: https://web.gin.g-node.org/gaalzs/SZEM.

## Ethics Statement

The studies involving human participants were reviewed and approved by Joint Psychological Research Ethics Committee (EPKEB, Hungary). The participants provided their written informed consent to participate in this study. Written informed consent was obtained from the individual presented on Figure 1 for the publication of any potentially identifiable images or data included in this article.

## Author Contributions

ZG, BN, DF, and IC designed the study. BN collected and analyzed the data. ZG, BN, and IC wrote the article. All authors contributed to the article and approved the submitted version.

## Conflict of Interest

The authors declare that the research was conducted in the absence of any commercial or financial relationships that could be construed as a potential conflict of interest.

The reviewer MZ declared a shared affiliation, though no other collaboration, with one of the authors BN to the handling editor.
